# Structural Model of the ETR1 Ethylene Receptor Transmembrane Sensor Domain

**DOI:** 10.1038/s41598-019-45189-w

**Published:** 2019-06-20

**Authors:** Stephan Schott-Verdugo, Lena Müller, Elisa Classen, Holger Gohlke, Georg Groth

**Affiliations:** 1 Institute for Pharmaceutical and Medicinal Chemistry, Heinrich Heine University Düsseldorf, Düsseldorf, Germany; 2 Centro de Bioinformática y Simulación Molecular (CBSM), Facultad de Ingeniería, Universidad de Talca, Talca, Chile; 3 Institute of Biochemical Plant Physiology, Heinrich Heine University Düsseldorf, Düsseldorf, Germany; 4 Present Address: Institute of Hydraulic Engineering and Water Resources Management, RWTH Aachen University, Aachen, Germany; 5 John von Neumann Institute for Computing (NIC), Jülich Supercomputing Centre (JSC) & Institute for Complex Systems - Structural Biochemistry (ICS 6), Forschungszentrum Jülich GmbH, Jülich, Germany; 6 Bioeconomy Science Center, Forschungszentrum Jülich GmbH, Jülich, Germany

**Keywords:** Biophysical chemistry, Plant signalling, Computational models

## Abstract

The structure, mechanism of action and copper stoichiometry of the transmembrane sensor domain of the plant ethylene receptor ETR1 and homologs have remained elusive, hampering the understanding on how the perception of the plant hormone ethylene is transformed into a downstream signal. We generated the first structural model of the transmembrane sensor domain of ETR1 by integrating *ab initio* structure prediction and coevolutionary information. To refine and independently validate the model, we determined protein-related copper stoichiometries on purified receptor preparations and explored the helix arrangement by tryptophan scanning mutagenesis. All-atom molecular dynamics simulations of the dimeric model reveal how ethylene can bind proximal to the copper ions in the receptor, illustrating the initial stages of the ethylene perception process.

## Introduction

Plant hormones regulate diverse aspects from growth and development to biotic and abiotic responses throughout the lifespan of plants. Signaling pathways and receptors have been identified to molecular detail for several of these signaling molecules, providing a basic integrated understanding on the mechanisms of hormone actions. High resolution structural information on receptors such as the brassinosteroid receptor BRI1^1,2^ or abscisic acid receptor PYR1/PYL1^3,4^ has been instrumental to understand receptor activation mechanisms^5,6^ or to identify small molecule mimetics activating receptors and related plant responses^7^. However, for most plant receptors such a level of structural and mechanistic understanding is still lacking.

Fruit ripening, senescence, and decay are highly relevant agronomical processes regulated by the plant hormone ethylene. Ethylene is perceived by a receptor family composed of ETR1, ERS1, ETR2, ERS2 and EIN4 in *Arabidopsis*^8^, which, in their functional state, form dimers and higher-molecular weight oligomers at the ER membrane^9^. These receptors show similarity to bacterial two-component histidine kinase receptors. All members of the receptor family have a similar overall modular structure composed of an N-terminal transmembrane sensor domain (TMD), a GAF domain in the middle portion, and a catalytic transmitter domain at the C-terminus. In receptors ETR1, ETR2, and EIN4, this basic structure is complemented by a C-terminal receiver domain. The presence of a copper cofactor in the TMD, likely in the +1 oxidation state^10^, was shown to be essential for ethylene binding and receptor function^11,12^. Yet, the copper stoichiometry in the functional dimer is still a matter of discussion. Bleecker, *et al*.^13^ consider one Cu^+^/monomer, while Rodriguez, *et al*.^10^ favor one Cu^+^/dimer. At present, the exact output of the receptors and mechanism of intramolecular and intermolecular signal transfer to further downstream elements in the ethylene signaling network is still obscure.

High-resolution structure information on the receptor is expected to solve this puzzle. Until today, crystal structures and low resolution SAXS models of the cytoplasmic part of ethylene receptors ERS1 and ETR1 excepting the GAF domain have been obtained^14,15^. This has allowed to obtain a model of the whole cytosolic domain, including the GAF, dimerization, catalytic, and receiver domains (residues 158 to 738, see Fig. 1 in ref.^14^); yet, this model lacks the connection to the transmembrane domain, and the transmembrane domain itself. In addition, mutational studies have contributed to a structure-function understanding. In that respect, most structural information comes from the studies of Wang, *et al*.^16^. Three main classes of *loss-of-function* mutants were identified, showing different levels of ethylene binding, signal transmission, or intrinsic activity. However, the structure of the TMD bearing the ethylene binding site has remained elusive. The current development of *ab initio* protein folding tools allows one to predict the structure. Such tools have been used for modelling, e.g., the N-terminal portion of the human dopamine transporter^17^ and the human alkylglycerol mono-oxygenase^18^. A notable application is the structure prediction of the human zinc transporter hZIP4^19^, for which mainly coevolutionary information was used. The thus obtained structure was later shown to be highly similar to a crystalized homolog^20^.

**Figure 1:**
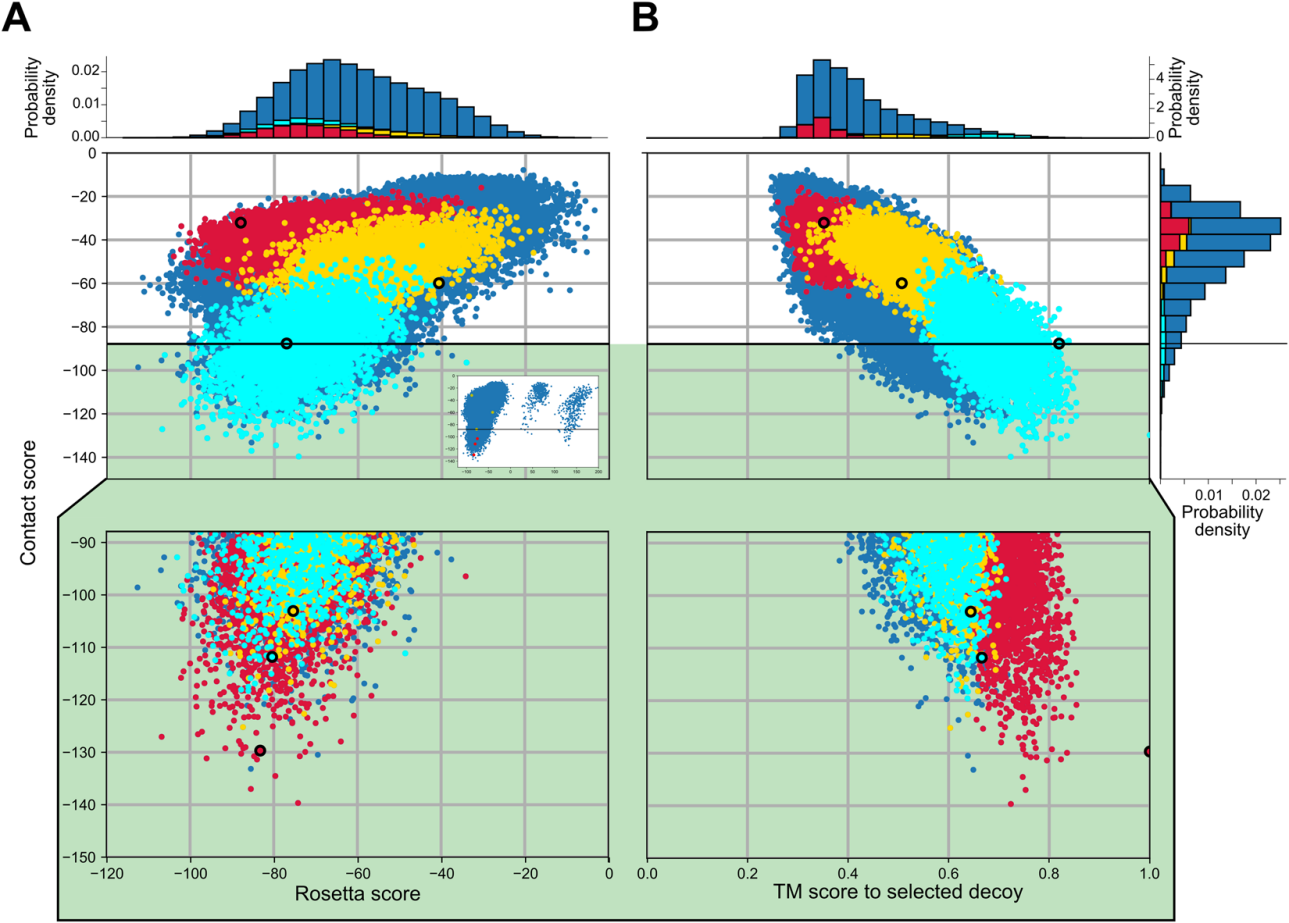
Distributions of Rosetta scores (membrane centroid score, **A**) and TM-scores with respect to the selected decoy (**B**) versus the calculated Contact score of the generated models. There is a positive correlation between the Rosetta score and the Contact score (*R*^2^ = 0.25, *p* < 0.001) for models with a negative Rosetta score (*R*^2^ = 0.01 for the complete distribution). The horizontal line demarks the −2 z-score threshold used with respect to the Contact score to filter out the worst scoring models; selected structures are shown on green background as a zoom. The models were structurally clustered in three groups pre- and post-score filtering, with the structures corresponding to the first, second, and third cluster shown in red, yellow, and cyan, respectively. The centroid models of the identified clusters (Fig. 2) are highlighted with a thick outline. The inset in panel A shows the complete distribution, including outliers.

Following the same principles as for hZIP4, we generated the first structural model of the transmembrane sensor domain of ethylene receptor ETR1 (ETR1_TMD) and a ETR1_TMD/Cu dimer model by integrating *ab initio* structure prediction and coevolutionary information to drive the model selection process according to the available experimental information. In addition, to refine and independently validate the obtained structural model, we determined protein-related copper stoichiometries experimentally on purified receptor preparations and explored the helix arrangement in the TMD using tryptophan scanning mutagenesis. The models obtained by this pipeline allow deriving experimentally testable hypotheses as to how ethylene accesses the copper cofactor, how copper is transferred to the TMD, and how ethylene binding leads to downstream signaling.

## Results

### Structural model of the transmembrane sensor domain ETR1_TMD

**Figure 2:**
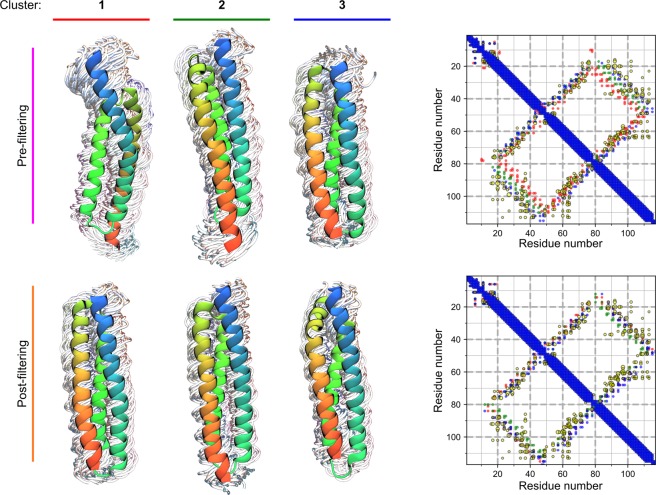
Clustered conformations and average contact maps for the generated decoys pre- (top) and post- (bottom) filtering according to the Contact score. The centroid of each cluster is shown in a cartoon representation, overlaid over every tenth other structure in the cluster, shown as wires. The structures are colored blue to red, starting from the N-terminal portion. The contact map for each set of clustered conformations are overlaid on the right, following the color scheme shown on top of every cluster to the left. Additionally, the MetaPSICOV contact predictions are shown as yellow dots. The size of the dots reflects the confidence assigned by the method. Before filtering, the clusters show different orientations with respect to helix three, as visible in the different conformations and on the contact map (residues 20–40 contacting 80–100). Contacts of the right-handed configuration of cluster 1 deviate the most from the MetaPSICOV contact predictions, and such configurations are removed by the filtering.

Currently, no homologs of ETR1 with an experimentally resolved structure are available. A search for structural templates in the Protein Data Bank resulted in structures with sequence identities below 15%, which would likely result in an imprecise structural model of ETR1 by homology modeling (see Supplementary Results). Hence, the structure of the TMD of ETR1 was modeled using the *ab initio* Rosetta protocol *membrane_abinitio2*, and further validated by filtering resulting structural models with co-evolutionary methods. A flowchart of the whole process can be found in the Supplementary Information (SI Fig. 6). The secondary structure and transmembrane topology predictions from the methods PSIPRED and CCTOP, respectively, were used in the model building process, and they agree on three TM α-helices for the ETR1_TMD, as previously predicted^16^ and shown for the close homolog ERS1^21^ (SI Fig. 2). The distribution of the 100,000 models generated shows that > 99% of them have negative, i.e., favorable, Rosetta scores (Fig. 1). The models were clustered with *Calibur*^22^, with an estimated C_α_ atom RMSD of residues 15 to 117 ranging from 1.1 to 22 Å. All clustered models are located in the membrane, as evaluated by the orientation of each model with respect to the “MEM” coordinates printed by the score_jd2 protocol; the evaluation was done visually in that it was assured that all models are oriented along the membrane normal and are embedded in the membrane slab. The representative structures of the three clusters obtained (Fig. 2, top row) reveal two helix arrangements with different handednesses of the helix bundle. To discriminate between these two potential solutions, coevolutionary residue-residue contact predictions from MetaPSICOV^23^ were used to rescore the models. The third largest cluster fulfills the predicted contacts best, as seen in the overlap of the predictions to the contact map (Fig. 2, top row, blue over yellow dots in the contact map) and given by a much more favorable average Contact score of −88, compared to −36 and −49 for the most and the second most populated clusters, respectively. Filtering the generated models for structures that fulfill the contact predictions the best removes all configurations with a right-handed helical bundle from the pool of 100,000 models, leaving 5,217 structures that only differ in the relative orientation of the helices and slightly in the positioning of the third helix with respect to the second (Fig. 2, compare top and bottom conformations and contact maps).

After clustering of the filtered models, each cluster has a quadratic mean of the pairwise RMSD, < RMSD^2^ > ^1/2^, of 3.1 to 3.2 Å^24^ and a distance in C_α_ atom RMSD of 2.8, 2.8, and 4.0 Å between centroids of clusters 1 and 2, 2 and 3, and 1 and 3, respectively. Both measures indicate the precision of our models^25^. The centroid of the most populated cluster (representing 40% of the filtered structures) was selected for further analysis (Fig. 2, bottom left and SI Fig. 4A). Most of the other models that have a favorable Contact score also have a TM score to that selected model of 0.5 or higher; the TM score is a measure analogous to RMSD, but less sensitive to local variations, and is bound between 0 and 1, where 1 relates to an identical 3D structure, indicating that these models have the same fold^26^ (Fig. 1). The selected model (SI Fig. 4A) was further refined with the Rosetta protocol *relax* to incorporate side chains^27^.

### Stoichiometry and affinity of copper(I) binding to ETR1_TMD

**Figure 3:**
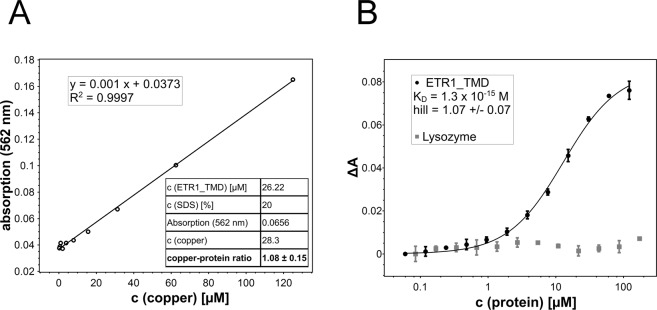
Copper-binding stoichiometry (**A**) and binding of copper(I) by the ETR1 transmembrane sensor domain (**B**). (**A**) Calibration curve of different BCA_2_-Cu(I) concentrations used to determine the copper concentration released from the protein (28.3 µM). Stoichiometry of copper-loaded ETR1 was determined by denaturing purified ETR1_TMD (26.22 µM) previously saturated with copper(I) by adding the detergent SDS at 20% (w/v) and heating the sample to 95 °C. The chromophoric copper chelator BCA (2 mM) was added, and absorption at 562 nm monitored. The table shown in the inset summarizes protein and copper concentrations of the experiment corresponding to a copper:protein molar ratio of 1.08:1. (**B**) Purified ETR1_TMD was titrated to BCA_2_-Cu(I) complex at concentrations from 122–0.06 µM. Binding of the metal ion was monitored spectrophotometrically by measuring absorbance of the purple BCA_2_-Cu(I) complex at 562 nm (black points). From the binding curve a dissociation constant K_D_ = 1.3 × 10^−15^ M and a Hill coefficient *h* = 1.07 ± 0.07 were calculated for copper binding to ETR1_TMD. The non-copper binding protein lysozyme (grey squares) was used as negative control. All measurements were run in triplicates.

To generate an **ETR1_TMD/Cu dimer** model (see below), the stoichiometry of ETR1_TMD with respect to the copper cofactor needs to be established. Previous studies have shown that the receptors sensing the plant hormone ethylene require monovalent copper ions in their transmembrane sensor domain in order to bind their ligand. Still, the number of copper ions per dimer forming the basic functional unit of the receptors is not clear. To clarify the stoichiometry of metal binding, purified ETR1_TMD was saturated with monovalent copper. To this end the purified sensor domain was incubated with saturating concentrations of the chromophoric copper chelator BCA, a water-soluble ligand that stabilizes the metal ion in the monovalent oxidation state. Transfer of the copper ion from the intensely purple-colored BCA_2_-Cu(I) chelate complex to the purified ETR1 sensor domain was monitored directly by absorption spectroscopy. Complete saturation of the copper binding sites in the receptor was achieved when no further change in the deeply purple-colored solution was detectable. Then, excess BCA_2_-Cu(I) chelate was removed by gel filtration, and the copper bound to the ETR1_TMD was released from the protein by chemical and thermal denaturation using the harsh detergent and amphipathic surfactant sodium dodecyl sulfate (SDS). The released metal ions were recomplexed by adding BCA, and the related copper concentration of the solution was determined by comparing the observed absorption at 562 nm to standard concentrations of BCA_2_-Cu(I). Figure 3A shows the calibration curve used for quantification. From these studies a metal-to-protein stoichiometry of one copper ion per ETR1_TMD was obtained (see table inserted in Fig. 3A).

Since monovalent copper promotes highly reactive oxygen species, the free copper concentrations in biological systems is kept extremely low due to high affinity binding to specific proteins, in order to avoid toxic effects. To determine the *in vitro* copper-binding affinity of ETR1_TMD, the purified sensor domain was titrated with Cu(I). As for the experiments resolving the copper stoichiometry of ETR1, the metal ion was stabilized in the monovalent oxidation state by BCA. Reduction in absorbance at 562 nm observed at increasing protein concentration was used to follow concentration-dependent copper binding to the receptor and to determine copper affinity of the purified TMD. Figure 3B shows the corresponding absorption changes as a function of the protein concentration added. The dissociation constant *K*_D_^1/2^ = 1.3 × 10^−15^ M (eq. 4) obtained from the metal-titration curve confirms the capacity of the purified ETR1 sensor domain to bind copper at very low concentrations, as previously reported for copper chaperones in living cells^28^. Under the same conditions, no changes in absorption and, thus, no copper binding was observed with the non-copper(I) binding protein lysozyme upon titration with the monovalent cation (also see Fig. 3B).

To confirm our stoichiometry analysis, titration data was fitted to the Hill binding model^29^. In this model, the stoichiometric coefficient *h* provides a measure for the cooperativity between binding sites and, in the extreme case of strict cooperativity, i.e., if all ligands bind at once (all-or-nothing binding), reflects the number of ligand binding sites on a protein. In this context, the Hill coefficient *h* = 1.07 ± 0.07 obtained from a fit of the titration data shown in Fig. 3B implies simple, non-cooperative copper binding at the ETR1 transmembrane sensor domain (lower bound of potential sites). In the case of strict binding cooperativity, this figure reflects a metal-to-protein stoichiometry of one copper ion per monomer (upper bound of potential sites) as obtained by direct analysis of the purified ETR1 sensor domain (Fig. 3B).

### Structural model of the ETR1_TMD/Cu dimer

**Figure 4:**
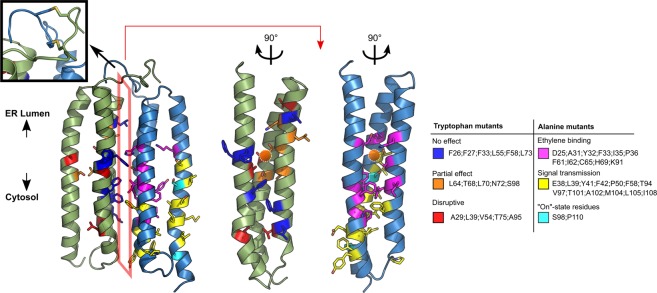
Dimer model of the ETR1_TMD. On the structure, the positions where tryptophan mutants were generated (left monomer) and where previous loss-of-function mutations were found^16^ (right monomer) are denoted by the color code on the right, with a list of the residues found in each class. The upper left inset shows the disulfide bridges included during the refinement of the protein. The orange spheres show the putative copper binding site in proximity to residues C65 and H69. The orange polygon indicates the interface. The two structures on the right represent the interface in an “open book” representation. Tryptophan mutants of residues that are pointing towards the monomer bundle core are disruptive (red) or are partially disruptive (orange), while mutations of residues pointing towards the dimer interface of the model (blue) showed no effect on the alpha-helical content, as shown in Fig. 5. Residues that are closer to the protein center and in proximity to the putative copper binding site are essential for ethylene binding (magenta), while residues farther away and closer to the cytosolic portion are responsible for the signal transmission (yellow). Residues shown to be relevant to maintain the protein in an active state are displayed in cyan.

From the generated ETR1_TMD model, and the determined copper stoichiometry, a dimeric model of ETR1_TMD/Cu can now be generated. For generating this model, the TMD model was docked using HADDOCK. Coevolutionary signals (Fig. 2 and SI Fig. 3), knowledge about low-lipophilicity regions (SI Fig. 2B), and the notion that the metal binding site should be shielded from the solvent^30^ were used as information to select the interacting interface; all this data suggests that the interface involves H2, while H3 is more membrane-exposed, indicating H1/H2 as the proper interface. The representative dimer model obtained from clustering after refinement by replica exchange MD simulations is shown in Fig. 4. The structure consists of an almost symmetrical arrangement of the previously modelled left-handed monomers. The N-terminal part displays the disulfide bonds in-between chains, formed by residues 4 and 6, respectively. The putative copper binding sites, composed of residues C65 and H69, are buried in the dimerization interface, as previously mentioned and suggested^16^; in the monomer, they would be exposed to the membrane environment. Based on the experimentally determined metal-to-protein stoichiometry of ETR1 (Fig. 3), two copper ions were added in subsequent steps in proximity to S_γ_ of C65 and N_δ_ of H69, considering that these residues are relevant for copper binding (Rodriguez, *et al*.^10^). The C-termini of the dimer are at opposite sides of the dimeric configuration, which would leave enough room for residues connecting the transmembrane domain and the cytoplasmic GAF domain. The obtained dimer model is supported by loss-of-function mutations according to which mainly H1 and H2 are involved in the binding of ethylene^16^ (for further details see Discussion section). These mutations form a “layered” spatial arrangement of residues, with relevant ethylene-binding residues close to the putative copper binding site and residues relevant to signal transmission close to the third helix/cytoplasmic region. Interestingly, the model shows residues S98 and P110, both of which seem to be essential for the correct transmission of the ethylene binding signal^16^, with the former pointing towards the copper binding site, while the latter generates a kink in the C-terminal portion of helix 3.

Based on the structural model, positions 26, 27, 29, 33, 39, 54, 55, 58, 64, 68, 70, 72, 73, 75, 95, and 98 were considered non-solvent/membrane exposed and good candidates for tryptophan scanning experiments; their mutation to tryptophan is expected to distort the structure as the relative solvent accessibility is reduced in the model ensemble (see also below).

### Experimental validation of the structural model by tryptophan scanning mutagenesis

**Figure 5:**
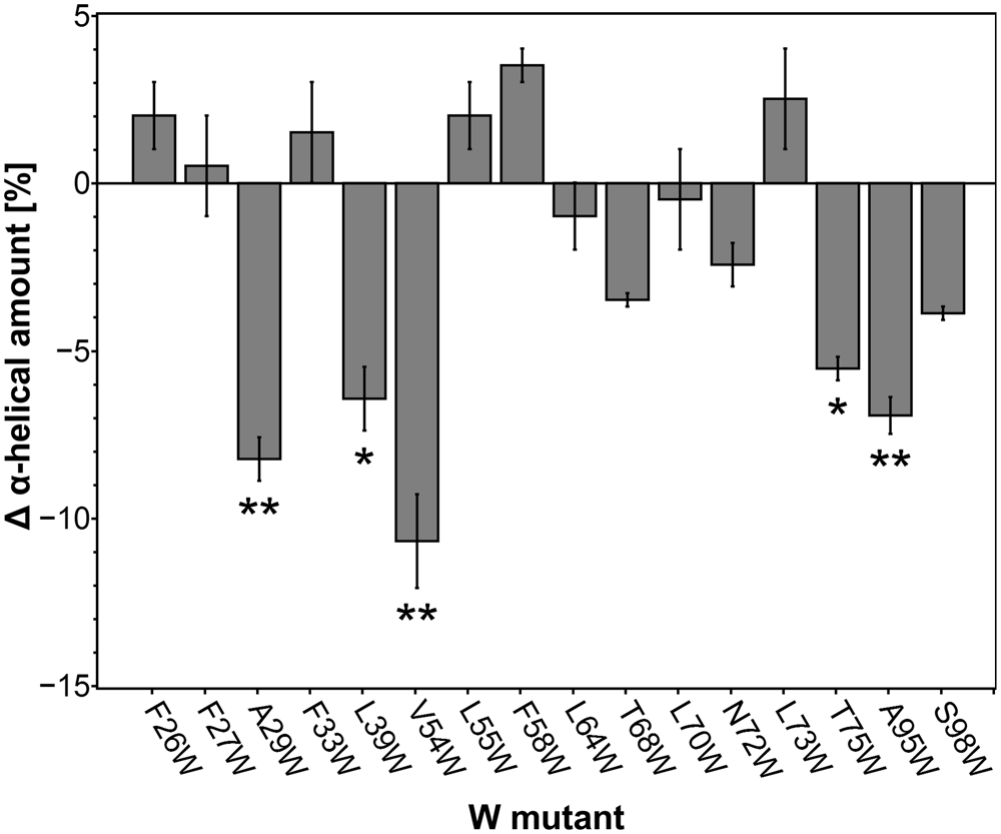
Changes in overall α-helical content in ETR1 by tryptophan substitution. Secondary structure content of purified full-length ETR1 tryptophan mutants was determined by CD spectroscopy. Changes in α-helical content related to individual tryptophan mutants are shown as deviation from the tryptophan-free background mutant ETR1^W7X^. All measurements were taken in triplicates. Mean and standard deviation are shown (*P ≤ 0.05; **P ≤ 0.02).

To validate the ETR1_TMD/Cu dimer model, tryptophan variants were generated. In the past tryptophan substitution mutagenesis has been applied as a useful tool to identify the relative arrangement and orientation of transmembrane helices in membrane proteins^31,32^. In this approach the large and moderately hydrophobic tryptophan side-chain is introduced at different positions of a transmembrane helix. Substitutions are tolerated at positions facing the membrane only, whereas introduction of tryptophan residues at helix-helix interfaces show disruptive effects on protein function or structure. Making use of this approach, ETR1 tryptophan mutants were generated based on predictions from the generated structural model of the ETR1_TMD/Cu dimer, and CD spectra of the variants were measured. From there, the α-helical amount of each mutant was computed. The tryptophan-free version of the full length ETR1 (ETR1^W7X^) shows an α-helical amount of 33%. Some of the other mutants show similar values, e.g., F27W (34%), F33W (35%), L64W (32%), and L70W (33%) (see Fig. 5, Supplemental Information Table 2, and Fig. 4). However, exchange of amino acids at positions 29, 39, 54, 75, and 95 to tryptophan show a significant decrease in α-helical amount to 22–27%. A lower amount in this value compared to the ETR1^W7X^ was used as an indicator for changes in protein structure due to specific tryptophan insertion.

### Molecular dynamics simulations of free ethylene diffusion show preferential occurrence of ethylene in the proximity of copper in the dimer model

In order to elucidate how ethylene can bind to the ETR1_TMD/Cu dimer model, molecular dynamics (MD) simulations of 1 μs length with an explicit membrane environment were performed (SI Fig. 4B). Ten independent replicates each were simulated, either containing 0.1 M ethylene in the water phase at the beginning of the simulation or not. Experimentally identified disulfide bridges between the protomers were included in the N-terminal region^33^. The trajectories show a moderate C_α_ atom RMSD drift with respect to the average structure (distribution maximum between 2 to 4 Å in all but one case (SI Fig. 5)), with all of the simulations reaching an apparent plateau after 500 ns. The copper ion was included with a 12-6-4 Lennard Jones non-covalent model, which has been shown to yield good coordination geometries with surrounding amino acids without additional restraints^34^. The ions remained bound to the sulfur atom of cysteinate C65 of each chain, interacted also with the non-protonated N_δ_ of H69, and showed infrequent interactions with D25 (SI Fig. 5). The overall coordination number between the protein and the copper ions fluctuates between 2 and 3, while there are 2 to 1 sites occupied by water molecules, yielding a total of 4 coordination sites. While the number of coordination sites may be biased by the used non-covalent model^34^, copper is coordinated in a similar manner in copper chaperones^35^.

**Figure 6:**
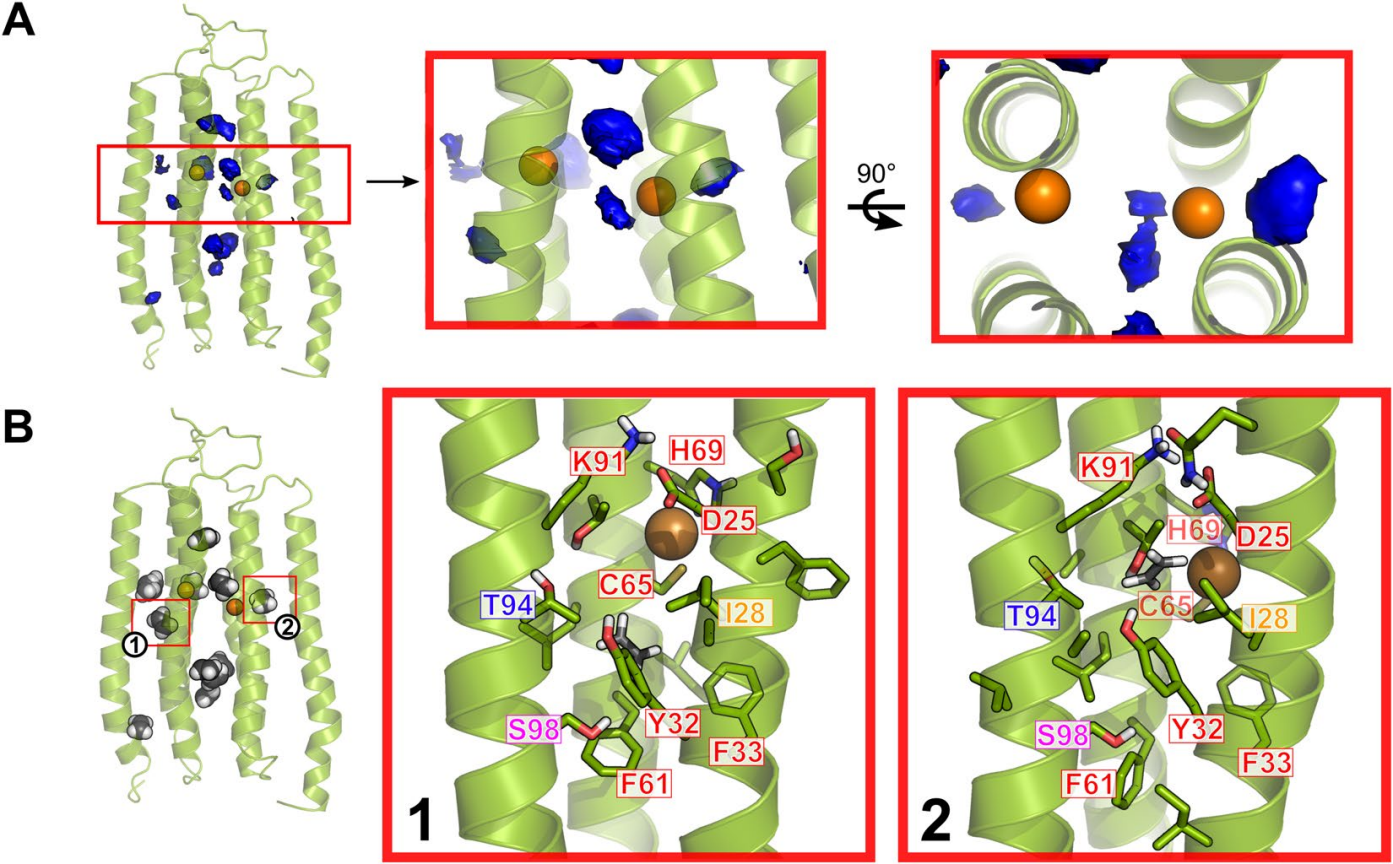
Putative ethylene binding sites identified during MD simulations. The average structure of the ETR1_TMD dimer over all replicate simulations is shown. (**A**) 3D propensity representation of the most probable locations of ethylene binding along all replicate simulations. The boxes highlight the portion close to the included copper ions, with a zoom on the blobs at a distance <5 Å. (**B**) Representative configurations of identified ethylene clusters. The clusters enclosed in the red boxes are at a distance <5 Å to the copper ions, and are shown in detail on the right. The residues labeled in red have been shown to be essential for ethylene binding, S98 (magenta) has a moderate effect, while T94 (blue) has no effect on binding, but is relevant for signal transmission. I28 has not been tested. Mutation information from Wang, *et al*.^16^.

According to the preparation of the simulation system, ethylene starts in the water phase but rapidly enters the membrane, and from there the ETR1_TMD (see also below), yielding a distribution with the maximum located in the central plane of the membrane (SI Fig. 5). A 3D histogram shows that ethylene has a higher propensity to bind within the TMD than the membrane, and it does so in particular regions of the TMD (Fig. 6A); these results are confirmed by a cluster analysis (Fig. 6B). Ethylene binding and unbinding occurs from the center of the membrane to the accessible binding site(s) in the protein; an example path is shown in SI Fig. 6B and C. That ethylene binding to the protein is almost in equilibrium is suggested by rapid and repeated binding/unbinding events (SI Fig. 6A) and the fact that ethylene binds similarly to both monomers (Fig. 6B), although no symmetry restraints were imposed during the MD simulations.

Three of the identified ethylene binding sites are at a distance < 5 Å to the copper ions, with one being in between the two ions, while the other two are located in the center of each monomer bundle (Fig. 6). These putative ethylene binding regions are close to the central plane of the membrane, as are the locations of the copper ions (SI Fig. 5). Residues lining ethylene binding sites close to the copper ions (Fig. 6B) are conserved (SI Fig. 2). Of these residues, seven have been shown to be essential for ethylene binding to the protein (red labels; (Fig. 6B)), and S98 has been shown to affect the binding moderately (magenta label; (Fig. 6B))^16^. In turn, of the residues found to be essential for ethylene binding aside from C65 and H69, which also have been linked with copper binding, only I35, P36, and I62 were not identified, but are in immediate proximity to the centroids of the described clusters. In some cases, distances between a copper ion and the center of mass of ethylene < 3 Å were observed, which is in the range of the sum of van der Waals radii for carbon and copper and only ~1 Å larger than distances observed in organometallic complexes^36^. While this clearly demonstrates a close approach of ethylene to copper, smaller distances cannot be expected, because the force field approach used here to model ethylene-copper interactions does not incorporate electron exchange between the binding partners, as expected from the Dewar-Chatt-Duncanson model of ethylene binding^37^.

## Discussion

In this study, we describe the first structural model of the transmembrane sensor domain of the plant ethylene receptor ETR1 at the molecular level. The structure reflects the functional dimeric state of the receptor with the bound copper cofactor, which was previously shown to be essential for ethylene binding and perception. Experimental analysis of purified ETR1 revealed a metal-to-protein stoichiometry of 1:1 and a dissociation constant (*K*_D_^1/2^, eq. 4) of 1.3 × 10^−15^ M for the monovalent copper cofactor.

Evidence for the 1:1 stoichiometry was obtained in two ways, using in both cases BCA as reporter and copper(I)-chelating system. First, the ETR1 sensor domain was saturated with the metal ion. Monovalent copper bound to the receptor then was released by denaturation of the protein, recomplexed with BCA, and determined spectrophotometrically. The measured copper concentration was then related to the concentration determined for the purified ETR1_TMD to obtain the molecular metal-to-protein ratio. In a second approach, the stoichiometry was deduced from the Hill coefficient of the binding isotherm obtained by titration of the soluble BCA_2_-Cu(I) chelate to increasing concentrations of the purified ETR1 sensor domain, assuming that the system shows strict cooperativity (all-or-nothing binding). Such behavior has been reported previously for various protein-ligand systems such as receptor interactions in tyrosine kinase ErbB-3/ErbB-2^38^, proton trapping in chloroplast ATPase^39^, or proton release in violaxanthin deepoxidase^40^. At first glance, the experimentally derived stoichiometry of one copper per monomer found in this work is contrary to earlier studies, which, based on the binding of radio-labeled ethylene in the presence of copper, proposed a stoichiometry of one copper per receptor dimer^10^. However, later work on this topic^41^ conceded that potentially not all receptors were active and capable of binding ethylene in this earlier study. Based on this boundary, the authors proposed an alternative model where each ETR1 receptor contains more than one copper ion per active receptor dimer - each one capable of binding ethylene^41^. The experimentally derived stoichiometry and molecular model of our study fit well to this idea.

The femtomolar affinity of copper towards the purified ETR1 sensor domain conforms well to the high metal affinities of 1.6 to 4.5 × 10^−11^ M reported for the metal binding domains of Menkes copper-transporting P_1B_-type ATPases - a homolog of plant RAN1^42^ – or the soluble chaperone Atox1^28^, which may be involved in copper transfer to the receptor at cellular conditions. Moreover, the higher copper affinity of the ETR1 sensor domain and the thermodynamic gradient to the above-mentioned cellular copper delivery systems may assure copper routing from the chaperons to the receptor target and thus an efficient net copper flux among these sites. Note, however, that these values are apparent affinities of purified proteins, which may change for membrane-integrated receptor dimers or receptor clusters at physiological conditions.

Generating a structural model of the transmembrane domain of ethylene receptors has been hampered so far by the lack of close homologs with known structure (see Supplementary Information). As an alternative, the Rosetta protocol for membrane protein modeling has been widely used for *ab initio* structure prediction^17,18^. The accuracy of protein structural models obtained by *ab initio* techniques is markedly improved by including co-evolutionary information into the model process^43^. A notable case was the structural model obtained for the human zinc transporter hZIP4^19^, which was later shown to be highly similar to a crystalized homolog^20^. Using the same principles as reported there, we resorted to the Rosetta membrane *ab initio* protocols and coevolutionary contacts to generate a structural model of the ETR1_TMD. The resulting large ensemble of structural models was screened with respect to coevolutionary residue-residue contact predictions. About 5% of the initial ensemble fulfilled the Contact score to values below −2 Z-score, showing an enrichment of models that favor the predicted contacts compared to the reduction shown for the hZIP4 modeling case^19^. The validity of the finally chosen model is further supported by the fact that measures of structural variability within a cluster of filtered models (<RMSD^2^>^1/2^) as well as between clusters of suitable models (C_α_ atom RMSD between centroid structures) are small and very similar (~3–4 Å). This indicates, first, a precision of the model comparable to structural deviations observed during molecular dynamics simulations started from crystal structures and, second, that no grossly different structural models have been generated with a likewise favorable Contact score.

For generating a dimer model from the left-handed helical bundle of three TM helices, we again used co-evolutionary information as restraints for protein-protein docking in addition to experimental knowledge on the shieldedness of the copper binding site^30^, information on the roles of C65 and H69 as copper-interacting and putatively copper-interacting residues, respectively^10,44,45^, and our own data on the copper:ETR1 monomer stoichiometry. The finding that there is one copper(I) per monomer suggests that there is one ethylene binding site per monomer as well, as the Dewar-Chatt-Duncanson interaction model involves one olefin per copper ion^11,12,36^. This also implies that the interplay between the monomers is probably necessary to transmit the signal to the C-terminal part of the receptor, rather than to provide a joint binding interface for ethylene.

The dimer model was validated by mutational analysis, both based on *loss-of-function* mutations previously described^16^ and tryptophan scanning mutagenesis performed here at sites identified from the dimer model. As to the former, residues involved in *loss-of-function* mutations form a layered arrangement around the copper binding site, which, first, indicates that in the structural dimer model, functionally relevant residues are in close proximity and, second, provides a suggestion on the mechanism how the receptor transmits the signal of ethylene binding to the cytosolic domain. The inner-center layer involves *loss-of-function* mutations on helices 1 and 2 (H1 and H2) relevant for (assisting in) binding of copper(I) and ethylene, while *loss-of-function* mutations on H1 and helix 3 (H3) closer to the cytosol are mainly involved in the transmission of the inhibitory signal to the rest of the protein. This suggests that a possible conformational change triggered by the binding of ethylene to the receptor is transmitted through H3 as a signal to the rest of the protein, thereby inhibiting it, while H1 and H2 provide support for the core of the binding site and might constitute the structural foundation against which the conformational change of H3 occurs. As to tryptophan scanning mutagenesis, tryptophan mutations that point to the hydrophilic core of the monomer according to our dimer model affected the helical content the most, as expected^31,32^. In contrast, mutations with a lower effect are either pointing to the membrane environment, again as expected, or the dimer interface. The latter seems surprising, but the incorporation of bulky residues at the interface might only affect the dimerization efficiency^19^ and not the overall α-helical content used here as a read-out for structural distortions.

We finally used the dimer model in all-atom explicit solvent/membrane MD simulations of free ethylene diffusion to probe if and how ethylene can access the copper cofactor. The MD simulations are converged with respect to ethylene binding from the central membrane slab to the ETR1_TMD dimer, as demonstrated by repeated and frequent binding and unbinding events and an almost symmetrical ethylene propensity within the protein. The MD simulations revealed one dominant binding site per monomer in the proximity of the copper ion. Notably, the location of this binding site is also in line with previously reported *loss-of-function* mutations^16^. The MD simulations furthermore reveal a prominent binding region between the monomers. However, accumulation of ethylene there may be due to a high concentration of ethylene used in the MD simulations and/or a lack of sufficiently high attraction between copper and ethylene. The latter results from missing charge transfer effects in the classical force field representation of intermolecular interactions used here^37,46^. In turn, this fact implicates that ETR1 itself provides sufficient affinity for ethylene, such that ethylene is attracted from the membrane already before the interaction with copper occurs. In line with this implication, the MD simulations also reveal that predominant paths of ethylene access and egress run parallel to the membrane and roughly at the center of the membrane slab (SI Figs 5C and 6).

Due to the only approximate representation of copper-ethylene interactions and the fact that no small-molecule model compound has been described yet in which cysteinate and histidine mimics, resembling the sidechains of C65 and H69, chelate Cu(I), we are currently unable to provide detailed insights into the exact binding geometry of ethylene at the copper ion in ETR1, the related electronic structure, and/or further influences of neighboring residues^36^. Providing such insights may also help answering the question how ETR1 manages to avoid unspecific reactions with dioxygen species^37^.

Still, the derived information can be used to speculate on the mechanism underlying the inhibition of ETR1 by ethylene. Residues S98 and P110 in H3 have been shown to be relevant to maintain the activity of the receptor, as mutations to alanine resulted in a permanently semi-inactivated state^16^. Our model shows that S98 is close to the putative ethylene binding site, while P110 generates a kink in the C-terminal region of H3. We now speculate that S98 is relevant to maintain H3 in a “receptor-on” conformation, which is perturbed due to the binding of ethylene, causing the inhibition of the receptor. The kink caused by P110, on the other hand, might be structurally required to correctly connect the transmembrane domain of ETR1 to the C-terminal part of the protein. The strain imposed by the lack of a helix breaking residue at this position might be enough to inhibit the whole receptor. Finally, binding of ethylene to copper might displace one of the coordinating residues and/or lead to a change in the coordination geometry of these residues. This change would be transmitted to the cytosolic domain. It has been previously shown that two component systems rely on amplifying the signal to transmit the message through the protein^47^. The binding of one ethylene molecule to each monomer might thus cause an even stronger signal, making a small coordination effect significant for the whole structure.

In summary, we have identified that ETR1 binds one copper(I) ion per monomer, and based on this information and coevolutionary signals, we built a dimeric *ab initio* model of the transmembrane sensor domain of ETR1 including copper(I). The structural model is supported by alanine and tryptophan scanning mutagenesis studies and reveals how ethylene accesses the center of each monomer bundle and can bind proximal to the copper(I) ions. From the model, we propose that ethylene binding perturbs interactions of H3 with the remaining receptor part, which leads to a signal towards the cytoplasmic domains that switches off the receptor.

## Materials and Methods

### *Ab initio* modeling with Rosetta

For modeling the transmembrane sensor domain of ETR1 of *Arabidopsis thaliana*, residues 1-117 (Uniprot code P49333) were used. The selection was based on the transmembrane topology prediction obtained by the meta approach CCTOP^48^ and on the secondary structure prediction from PSIPRED v4^49^ (both shown in SI Fig. 2A). The former was chosen as it averages via a weighted Hidden Markov Model results of ten state-of-the-art transmembrane topology predictors and has shown a significantly higher accuracy^48^. A search of protein templates was performed, resulting in a maximum sequence identity of 11.3% (Supplementary Information). As a consequence, the protein was modelled *ab initio*. For this, the exposure of the transmembrane portion of each predicted helix towards the membrane environment was evaluated with the LIPS algorithm^50^. Based on the secondary structure prediction, 3- and 9-mer fragments were generated by using the *make_fragments.pl* script included in Rosetta 3.6. These initial predictions were included into the modelling process, which was performed using the RosettaMembrane *membrane_abinitio2* protocol (Supplementary Methods), also included in Rosetta^27,51^. The modelling followed the developer recommendations^52^, generating 100,000 centroid models (structures without explicit side-chains). To evaluate the orientation of the models in the membrane with respect to the “MEM” coordinates printed by the score_jd2 protocol, it was assured visually that all models are oriented along the membrane normal and are embedded in the membrane slab. Contact predictions were calculated using MetaPSICOV with the default protocol^23^, generating an alignment of 447 homologs with an alignment depth of 3.8, which is in line with previously shown modelling scenarios^43,53^. As a result, 397 predicted contacts with a confidence > 0.1 were obtained. The contact predictions were transformed into Rosetta constraint format between the C_β_ atoms (C_α_ if glycine) to score the obtained models, using the following sigmoidal function:

1 $${\rm{Contact}}\,{\rm{score}}\,f(c,r)=c(\frac{1}{1+{e}^{-3(r-8)}}-1)$$

where *c* corresponds to the prediction confidence used as a weighting factor and *r* is the corresponding distance between C_β_ atoms in Ångstroms. This is the suggested way to score models in the GREMLIN contact web-server^54^. The sigmoid was centered at a distance of 8 Å, following the contact prediction convention. The models were scored during the folding process using a weight of 4, and were filtered using a z-score ≤ −2 with respect to the contact score, similar to what has been used before^19^, reducing the number of structures to 5217. To identify a representative structure of the ensemble, a clustering step was performed by using *Calibur*^22^. Contact maps for the decoys corresponding to each cluster were calculated and averaged by using a 8 Å threshold. The centroid of the largest identified cluster was used for further analysis and *relaxed* using the corresponding Rosetta protocol (Supplementary Methods) with membrane scoring and the RosettaMembrane *membrane_highres* scoring weights, generating 100 decoys with different side-chain configurations. The per-residue solvent accessibility of the models in the selected cluster was evaluated using FreeSASA^55^, and residues with low average accessibility were considered for tryptophan mutation analysis. To have a measure of the sampled model space and convergence of the filtered models, the final selected model was compared with the rest by using the template modeling score (TM score), a measure of structural similarity analogous to RMSD, but less sensitive to local variations, which is bounded between 0 and 1; values > 0.5 have been shown to reflect similar folds^56^.

### Dimer model generation

The decoy with the highest ProQM^57^ and high QMEANbrane^58^ scores was selected from the relaxed configurations. Both scores are model quality assessment scores for membrane proteins, which allow evaluating protein structures without knowing the target structure, and are derived from top-ranked methods in the CASP12 competition^59^. To generate a dimer structure, two copies of this decoy were docked together using HADDOCK^60^. Residues of the TM1/TM2 interface were selected to fulfil restraints between monomers. The rationale for selecting this interface was twofold: I) it has been proposed that copper ions bind to residues C65 and H69 on TM2^10^ and that residues chelating metal ions are less solvent accessible^30^; II) TM1 and TM2 have more helical surfaces with a low LIPS score and stronger coevolutionary signals between them considering homodimeric symmetry (SI Fig. 2). Both I) and II) have been shown to be predictive of interactions between monomers^61^ and, thus, were used to guide the docking with HADDOCK. Additional unambiguous restraints between residues E15 and K45 were included to promote a near-parallel and membrane-like orientation of the monomers. The first 15 unstructured aminoacids were removed to avoid sampling restrictions during the rigid docking phase, the DMSO model was used for the “water” refinement stage, and a C_2_ symmetry was enforced throughout the docking process. The generated models were scored with QMEANbrane as before, and the N-terminal residues were modelled with MODELLER^62^, including restraints between residues 4 and 6 of monomers, respectively, to represent experimentally shown disulfide bonds^33^.

The relative orientation between the transmembrane helices in the dimer was refined by using a replica exchange molecular dynamics protocol (REMD) in a GBSW^63^ implicit membrane model. The sampling of the relative orientation of the helices was enforced by imposing restraints on dihedral angles in portions of the protein with predicted secondary structure. It has been shown previously that refinement of models through molecular dynamics (MD) simulations is well suited for local structure refinement but can drift the model away from the real structure if no restraints are used^64^. The simulations were performed using charmm 41b2 with the included CHARMM22 GBSW parameters and run using the MMTSB Tool Set^65^ *aarex.pl* script. For this, 16 exponentially spaced replicas between 300 and 460 K were used, ensuring an exchange probability between 12 and 15% throughout the simulations. An exchange was tried every picosecond of simulation time, and each replica was run for 5 ns, making a total of 80 ns. The temperature was maintained with a Langevin thermostat with a friction coefficient of 5 ps^−1^. A membrane thickness of 30 Å, a switching length of 2.5 Å, and a surface tension coefficient of 0.005 kcal mol^−1^ Å^−2^ were used. The replica running at 300 K was clustered by using the DBSCAN clustering algorithm in CPPTRAJ^66^. The representative structure of the biggest cluster was selected for further MD simulations.

### Molecular dynamics simulations of the ETR1_TMD/Cu dimer model in the absence and presence of ethylene

The refined model was embedded into a DOPC:DOPE 3:1 membrane, resembling the major components of the plant endoplasmic reticulum membrane^67^, by using PACKMOL-Memgen^68^. A Cu^+^ ion was included per subunit (see below with respect to the stoichiometry) in between residues C65 and H69, and K^+^ and Cl^−^ were added as counterions in the solvation box. Ions were treated with a 12-6-4 non-bonded model^34^. The GPU particle mesh Ewald implementation from the AMBER18 molecular simulation suite^69^ with the ff14SB^70^ and Lipid17 (^71^ and Skjevik *et al*.^72^) parameters for the protein and the membrane lipids, respectively, were used; water molecules were added using the TIP3P model^73^. Ten independent MD simulations of 1 µs length were performed. Covalent bonds to hydrogens were constrained with the SHAKE algorithm^74^ in all simulations, allowing to use a time step of 2 fs. All analyses were performed using CPPTRAJ^66^.

Initially, the total potential energy was minimized by three mixed steepest descent/conjugate gradient minimizations with a maximum of 20,000 steps each. First, the initial positions of the protein and membrane were restrained, followed by a calculation with restraints on the protein atoms only and, finally, a minimization without restraints. The temperature was maintained by using Langevin dynamics^75^, with a friction coefficient of 1 ps^−1^. The pressure, when required, was maintained using a semi-isotropic Berendsen barostat^76^, coupling the membrane (x-y) plane. The equilibration started from the minimized structure, which was heated by gradually increasing the temperature from 10 to 100 K for 5 ps under NVT conditions, and from 100 to 300 K for 115 ps under NPT conditions at 1 bar. This was continued for 5 ns under NPT conditions, after which production runs were started using the same setup. Ethylene was parametrized with the general Amber force field (GAFF)^77^, and RESP charges^78^ were obtained from electrostatic potentials computed at the HF/6-31 G* level of theory with Gaussian 09^79^ and fitting with *antechamber*^80^. Ethylene was included at a concentration of 0.1 M in the water volume, and its diffusion was observed in an unbiased manner^81,82^.

### Cloning of ETR1 tryptophan mutants and ETR1_TMD

DNA coding tryptophan-free ETR1 (ETR1^W7X^) from plasmid pET16b-ETR1(ΔW)^83^ was cloned in vector pET15b. The resulting vector carrying ampicillin resistance and an N-terminal Hexahistidine-tag was used for cloning a set of ETR1 tryptophan substitution mutants. Mutants were either cloned by using mega primer built on mutagenesis primer or by round-the-horn site-directed mutagenesis^84^. pET16b_ETR1_TMD was cloned from pET16b-ETR1^85^ which differs from vector pET15b by an N-terminal Decahistidine-tag by round-the-horn site-directed mutagenesis. Oligonucleotides used by round-the-horn site-directed mutagenesis were phosphorylated at their 5′ ends. A table with all oligonucleotides used can be found in the supplemental information.

### Expression of ETR1 tryptophan mutants and ETR1_TMD in *E. coli* C43

Expression of ETR1 protein constructs was performed in 2YT media (1.6% (w/v) peptone, 1% (w/v) yeast extract, and 0.5% (w/v) NaCl) containing 100 µg/ml ampicillin for selection. For agar plates 1.5% (w/v) agar was added. Vectors pET15b or pET16b carrying the DNA sequence for ETR1 tryptophan mutants or ETR1_TMD, respectively, were individually transformed into the expression host *E. coli* C43 and incubated on agar plates at 37 °C. The main culture was inoculated with a preculture to an OD_600_ = 0.1. For ETR1 tryptophan mutant expression, cells were grown at 30 °C and 180 rpm, and expression was induced by adding 0.5 mM IPTG at an OD_600_ = 0.8. After 5 hours, cells were harvested by centrifugation at 7,000 g and 4 °C for 15 min, flash frozen in liquid nitrogen and stored at −20 °C. For expression of ETR1_TMD, 2% (v/v) ethanol was added to the media, and cells were grown at 30 °C and 180 rpm until OD_600_ = 0.4 was reached. Temperature was reduced to 16 °C, and protein expression was induced with 0.5 mM IPTG at an OD_600_ = 0.6. Cells were incubated for 20 h and harvested as described for ETR1 tryptophan mutant expression.

### Sequential fractionation and isolation of full-length ETR1 and ETR1_TMD

Cells were resuspended in buffer M (PBS, 10% (w/v) glycerol, 0.002% (w/v) PMSF, and 10 µg/µL DNaseI) and broken in a Constants Cell Disruption System (Constant Systems, Daventry, United Kingdom) at 2.4 kbar and 4 °C. The lysate was centrifuged at 14,000 g and 4 °C for 30 min. The supernatant was centrifuged again at 40,000 g and 4 °C for 30 min. The resulting pellet was resuspended in buffer M and centrifuged at 34,000 g and 4 °C for 30 min. Finally, the pellet was flash frozen in liquid nitrogen and stored at −80 °C.

### Denatured purification and renaturation of ETR1 tryptophan mutants

Membrane fractions of ETR1 tryptophan mutants were resuspended in buffer D (50 mM TRIS/HCl pH 8, 100 mM NaCl and 8 M urea) and stirred for 2 h at 37 °C before centrifugation at 100,000 g and RT for 30 min. The supernatant was loaded to a buffer D-equilibrated HisTrap FF column operated by an ÄKTAprime plus (both GE Healthcare Life Sciences) and purified by immobilized metal-ion affinity chromatography (IMAC) at 4 °C. The column was washed with buffer D containing 50 mM imidazole, and the protein was eluted with buffer D containing 500 mM imidazole. Protein fractions were pooled and concentrated to 1.5 ml, refilled to 15 ml with buffer D and again concentrated to a final concentration of 0.8 mg/ml. For renaturation 100 mM DTT was added to 0.5 ml protein solution (0.8 mg/mL), mixed with 10 ml buffer R (55 mM TRIS/HCl pH 8, 264 mM NaCl, 11 mM KCl, 0.1% (w/v) DDM, 1.1 mM EDTA, 10 mM DTT, and 0.002% (w/v) PMSF) and centrifuged at 229,600 g and 4 °C for 30 min. The protein solution was concentrated to 500 µl, buffer was changed to buffer C (50 mM potassium phosphate pH 7.5 and 0.05% (w/v) DDM), concentrated to a final concentration of 0.1–0.3 mg/mL for circular dichroism spectroscopy studies.

### Solubilization and purification of ETR1_TMD

Membrane fraction of ETR1_TMD was resuspended in buffer S (50 mM TRIS/HCl pH 8, 200 mM NaCl, 1.2% (w/v) FosCholine-16, and 0.002% (w/v) PMSF), stirred for 1 h at RT, and centrifuged for 30 min at 229,600 rpm and 4 °C. Protein was purified by IMAC. To this end, the supernatant was loaded to a HisTrap FF column equilibrated in buffer A (50 mM TRIS/HCl pH 8, 200 mM NaCl, 0,015% (w/v) FosCholine-16, and 0.002% (w/v) PMSF). After washing with 20 column volumes with buffer ATP (50 mM TRIS/HCl pH 8, 200 mM NaCl, 50 mM KCl, 20 mM MgCl_2_, 10 mM ATP, and 0.002% (w/v) PMSF), the column was washed with buffer A containing 50 mM imidazole. ETR1_TMD was eluted from the column with buffer A containing 250 mM imidazole and fractions containing the purified protein were concentrated to 2.5 ml. Buffer was changed to buffer A and protein was loaded to a Superdex 200 Increase 10/300 GL column previously equilibrated with buffer A (GE Healthcare Life Sciences) and further purified by size exclusion chromatography. Protein fractions containing the purified ETR1_TMD dimers were pooled, concentrated to a minimum final concentration of 200 µM and used for copper binding studies.

### Circular dichroism spectroscopy of ETR1 tryptophan mutants

CD measurements of ETR1 tryptophan mutants were performed using a *Jasco-715* spectropolarimeter (Jasco GmbH, Gross-Umstadt, Germany) and a cylindrical quartz cuvette (Hellma GmbH & Co. KG, Muellheim) with a path length of 1 mm and a volume of 200 μl. All measurements were performed at room temperature in buffer C at a protein concentration of 0.1–0.3 mg/ml. Spectra were recorded from 260–195 nm with a step resolution of 1 nm and a bandwidth of 2 nm. The scan speed was set to 50 nm/min, and 10 scans were accumulated. Secondary structure content of purified proteins was calculated from the spectra by Selcon3 and CONTINLL.

### Copper binding studies on purified ETR1_TMD

Protein-related copper stoichiometries were determined from ETR1_TMD samples saturated with BCA_2_-Cu(I). First, these were loaded on PD10 mini columns (GE Healthcare Life Sciences) to remove excess BCA_2_-Cu(I). Subsequently, protein concentration of the samples was determined by measuring absorbance at 280 nm in a TECAN plate reader Infinite M200 PRO using a Nano Quant plate (Tecan, Männedorf, Schweiz). For determination of protein-related copper stoichiometries, ETR1_TMD was denatured with SDS at a final concentration of 20% (w/v) and heated at 95 °C for 10 min resulting in the release of copper cofactor bound to the protein. To complex the copper released by the protein, the solution then was incubated for 10 min with 2 mM BCA. After incubation, absorbance at 562 nm was measured, and copper concentration in the sample was calculated from a standard curve obtained by measuring the absorbance of different concentrations of BCA_2_-Cu(I) at 562 nm^86^. All samples were run in triplicates.

For copper binding studies, ETR1_TMD was serially diluted from 122 µM to 60 nM in a transparent 96 well plate (Sarstedt/Nümbrecht) at a volume of 25 µL for each concentration. Then, an equivalent volume of 25 µl of a 1:10 dilution of BCA_2_-Cu(I) reagent (50 mM TRIS/HCl pH 7.5, 200 mM NaCl, 2.5 mM BCA, 1 mM CuCl, and 20 mM ascorbate) was mixed with each of the ETR1_TMD samples. Absorption of the purple BCA_2_-Cu(I) complex at 562 nm was measured in a TECAN plate reader Infinite M200 PRO (Tecan, Männedorf, Schweiz). Lysozyme - a non-copper(I) binding protein - was used as negative control in these measurements^87^. Based on previous studies recognizing homodimers as the minimum functional unit of the ethylene receptor family^88^ titration data of the purified TMD with BCA_2_-Cu(I) was fitted to a binding isotherm reflecting the following equilibrium:

2 $${({\rm{ETR}}1\_{\rm{TMD}})}_{2}+2{({\rm{BCA}})}_{2}-{\rm{Cu}}\to {({\rm{ETR}}1\_{\rm{TMD}})}_{2}-{\rm{Cu}}{({\rm{I}})}_{2}+4\,\mathrm{BCA}\,$$

which consists of the two partial reactions

2a $${({\rm{BCA}})}_{2}-{\rm{Cu}}({\rm{I}})\to {\rm{Cu}}({\rm{I}})+2\,{\rm{BCA}}$$

and

2b $${({\rm{ETR}}1\_{\rm{TMD}})}_{2}+2\,{\rm{Cu}}({\rm{I}})\to {({\rm{ETR}}1\_{\rm{TMD}})}_{2}-{\rm{Cu}}{({\rm{I}})}_{2}$$

The equilibrium constant for eq. 2 then is

3 $${K}_{R}=\frac{[{(ETR1\_TMD)}_{2}-Cu{(I)}_{2}]{[BCA]}^{4}}{[{(ETR1\_TMD)}_{2}]{[{(BCA)}_{2}-Cu(I)]}^{2}}=\frac{[{(ETR1\_TMD)}_{2}]{[Cu]}^{2}{[BCA]}^{4}}{{K}_{D{(ETR1\_TMD)}_{2}-Cu{(I)}_{2}}[{(ETR1\_TMD)}_{2}]{[{(BCA)}_{2}-Cu(I)]}^{2}}=\frac{1}{{K}_{D{(ETR1\_TMD)}_{2}-Cu{(I)}_{2}}{\beta }_{2}{([{(BCA)}_{2}-Cu(I)])}^{2}}$$

From eq. 3, the dissociation constant for eq. 2b, ${K}_{D{(ETR1\_TMD)}_{2}-Cu{(I)}_{2}}$, is calculated from *K*_R_ and previous estimates of the formation constant of BCA_2_-Cu(I), β_2_ = 2*10^17^ M^−2^ ^89^.

4 $${K}_{D{(ETR1\_TMD)}_{2}-Cu{(I)}_{2}}=\frac{1}{{K}_{R}\,{\beta }_{2}{([{(BCA)}_{2}-Cu(I)])}^{2}}=\frac{1}{1.5\times {10}^{-5}{M}^{2}\times 4\times {10}^{34}{M}^{-4}}=1.7x{10}^{-30}{M}^{2}$$

This value corresponds to a ${\rm{dissociation}}\,{\rm{constant}}\,{K}_{D{(ETR1\_TMD)}_{2}-Cu{{(I)}_{2}}^{1/2}}$ of 1.3 × 10^−15^ M per copper equivalent.

## Supplementary information

Supporting Information
