# Supplementary material for: Structural Model of the ETR1 Ethylene Receptor Transmembrane Sensor Domain
Source: Sci Rep. 2019 Jun 20;9:8869. doi: 10.1038/s41598-019-45189-w (PMC6586836; doi:10.1038/s41598-019-45189-w)
Supplement: Supplementary file 1 — Supporting Information [file 41598_2019_45189_MOESM1_ESM.pdf]

Supporting Information

Structural Model of the ETR1 Ethylene Receptor Transmembrane Sensor Domain

Stephan Schott-Verdugo[1,2,†], Lena Müller[3,†], Elisa Classen[3,4], Holger Gohlke[1,5,6,*], Georg Groth[3,6,*]

[1]Institute for Pharmaceutical and Medicinal Chemistry, Heinrich Heine University Düsseldorf, Düsseldorf, Germany
[2]Centro de Bioinformática y Simulación Molecular (CBSM), Facultad de Ingeniería, Universidad de Talca, 2 Norte 685, CL-3460000 Talca, Chile
[3]Institute of Biochemical Plant Physiology, Heinrich Heine University Düsseldorf, Düsseldorf, Germany
[4]Present address: Institute of Hydraulic Engineering and Water Resources Management, RWTH Aachen University, Aachen, Germany
[5]John von Neumann Institute for Computing (NIC), Jülich Supercomputing Centre (JSC) & Institute for Complex Systems - Structural Biochemistry (ICS 6), Forschungszentrum Jülich GmbH, Jülich, Germany
[6]Bioeconomy Science Center, Forschungszentrum Jülich, Jülich, Germany

*Corresponding Authors: Holger Gohlke; Georg Groth

Address: Universitätsstr. 1, 40225 Düsseldorf, Germany.

Phone: (+49) 211 81 13662 or 12822

E-mail: gohlke@uni-duesseldorf.de, groth@uni-duesseldorf.de

1 Supplementary Methods

a) membrane_abinitio2:

The *ab initio* folding was performed in batches of 100 structures across multiple computers using the following flags (bold text represents options/paths that have to be replaced appropriately):

```
-in:file:fasta FASTA_FILE
-seed_offset RANDOM_NUMBER
-in:file:spanfile SPAN_FILE
-in:file:lipofile LIPS4_FILE
-in:file:frag3 3MERS_FILE
-in:file:frag9 9MERS_FILE
-in:path:database DATABASE_PATH
-abinitio:membrane
-score:find_neighbors_3dgrid
-membrane:no_interpolate_Mpair
-membrane:Menv_penalties
-out:file:silent SILENT_FILE
-out:sf SCORE_FILE
-out:nstruct 100
-out:file:silent_struct_type binary
-mute all

-constraints
    -cst_file CONSTRAINT_FILE
    -cst_weight 4
```

b) relax:

The selected centroid model was relaxed to incorporate side chains using the following flags:

```
-in:file:s PDB_FILE
-in:file:spanfile SPAN_FILE
-in:file:lipofile LIPS4_FILE
-in:path:database DATABASE_PATH
-relax:thorough
-out:file:silent_struct_type binary
-membrane
-membrane:Membed_init
-score:weights membrane_highres.wts
-nstruct 100
-out:file:silent SILENT_FILE
```

c) score_jd2:

The predicted membrane orientation was obtained by rescoring with the following flags:

```
-in:file:silent SILENT_FILE
-in:file:spanfile SPAN_FILE
-in:file:lipofile LIPS4_FILE
-in:path:database DATABASE_PATH

-score:weights score_membrane
-score:find_neighbors_3dgrid
-membrane:no_interpolate_Mpair
-membrane:Menv_penalties
```

2 Supplementary Results

ETR1 transmembrane domain homolog search. The homolog search for the transmembrane domain of ETR1 was performed using the same N-terminal 117 residues as for *ab initio* modeling. For this, the threading module of TopModel was used [1]. The top three identified homologues were (sequence identity and protein in parenthesis) 2C12_A (11.3%, nitroalkane oxidase), 1EGD_A (9.7%, acyl-CoA dehydrogenase) and 1RX0_A (7.3%, isobutyryl-CoA dehydrogenase), none of which corresponds to transmembrane proteins. Even when a transmembrane protein could be modeled based on the hydrophobic core of a soluble protein, the additional low sequence identity[2] and the lack of homology urged us to sample the fold space by *ab initio* modelling.

MetaPSICOV and Rosetta constraint files. Files containing both constraints are accessible as separate files at https://uni-duesseldorf.sciebo.de/s/i0s6IKlZmw8v5Ef.

PDB file of the ETR1_TMD/Cu dimer. Coordinates of the dimer model depicted in Figure 4 are accessible as a separate PDB file at https://uni-duesseldorf.sciebo.de/s/i0s6IKlZmw8v5Ef.

3 Supplementary Figures

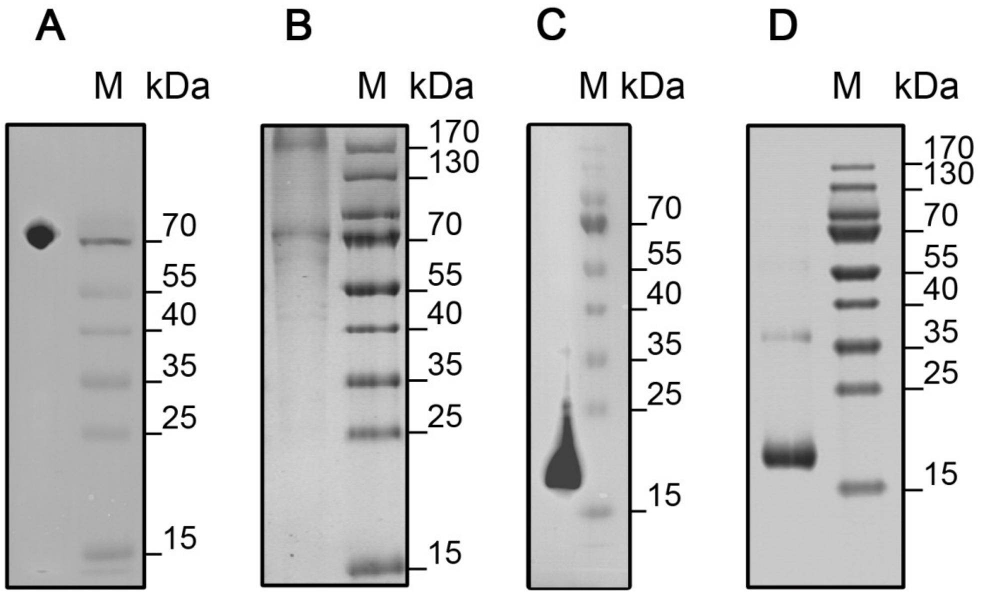

SI Figure 1. Expression and purification of ETR1 tryptophan mutants and ETR1_TMD. *E. coli* C43 (DE3) was transformed with a pET16b vector carrying the DNA sequence of ETR1 tryptophan mutants and ETR1_TMD, respectively. ETR1 tryptophan mutants (A) were expressed for 5 h at 30 °C and ETR1_TMD (C) was expressed for 20 h at 16 °C. For both expressions host cell extract was analyzed by western blotting. For protein detection an Anti-His antibody targeting the proteins deca-histidine tag was used. For purification from the bacterial host, ETR1 tryptophan mutants (B) were purified by IMAC and ETR1_TMD (D) was purified by IMAC and SEC. Analysis was performed by coomassie stained SDS-PAGE.

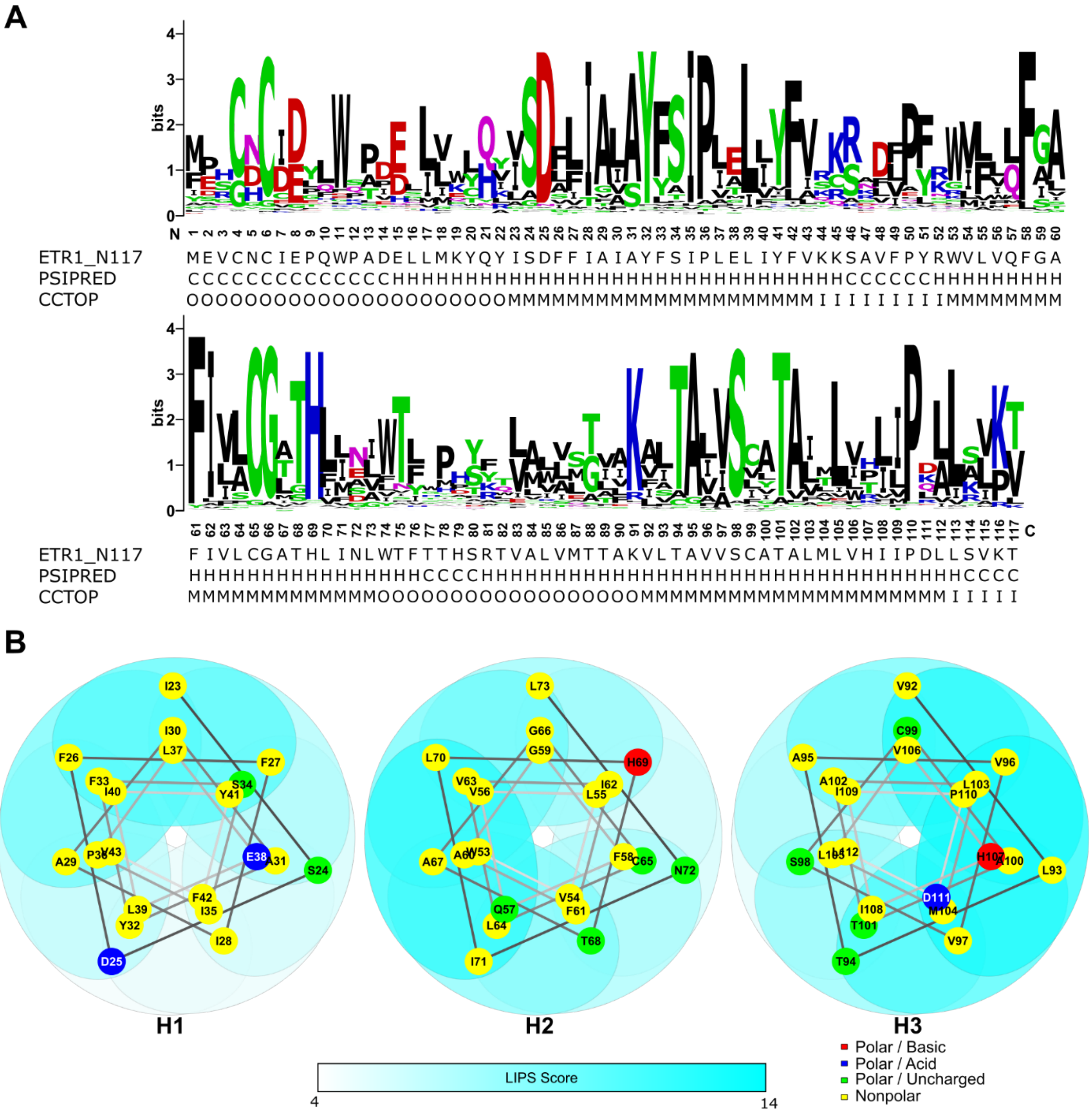

SI Figure 2. Analysis of modeled ETR1_TMD structure. A) Weblogo [3], PSIPRED secondary structure, and CCTOP transmembrane topology predictions for the modeled structure. C = coil; H = helix; O = extra-cytosolic region; M = transmembrane region; I = cytosolic region. B) Schematic representation of the LIPS score of the surfaces of the predicted helical transmembrane helices H1, H2 and H3. A higher LIPS score implies a higher propensity to be exposed to the membrane environment. The helical wheel representations were modified from NetWheels[4].

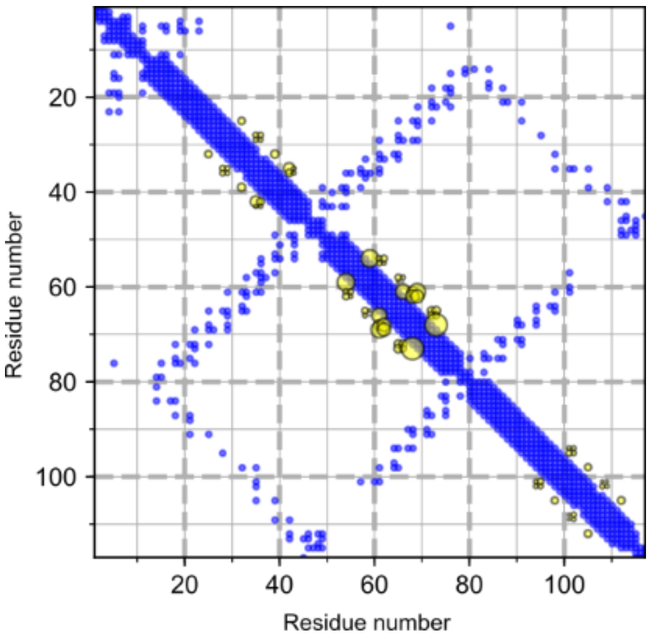

SI Figure 3. Coevolutionary signals on transmembrane helix surfaces with high LIPS score and at a sequence distance of 8 or less residues (yellow). These signals have been proposed as being indicative of dimerization interface. The signals are shown with a radius 30 times bigger than those shown in Figure 2 in the main text. The contact map of the selected monomer model is shown for reference (blue).

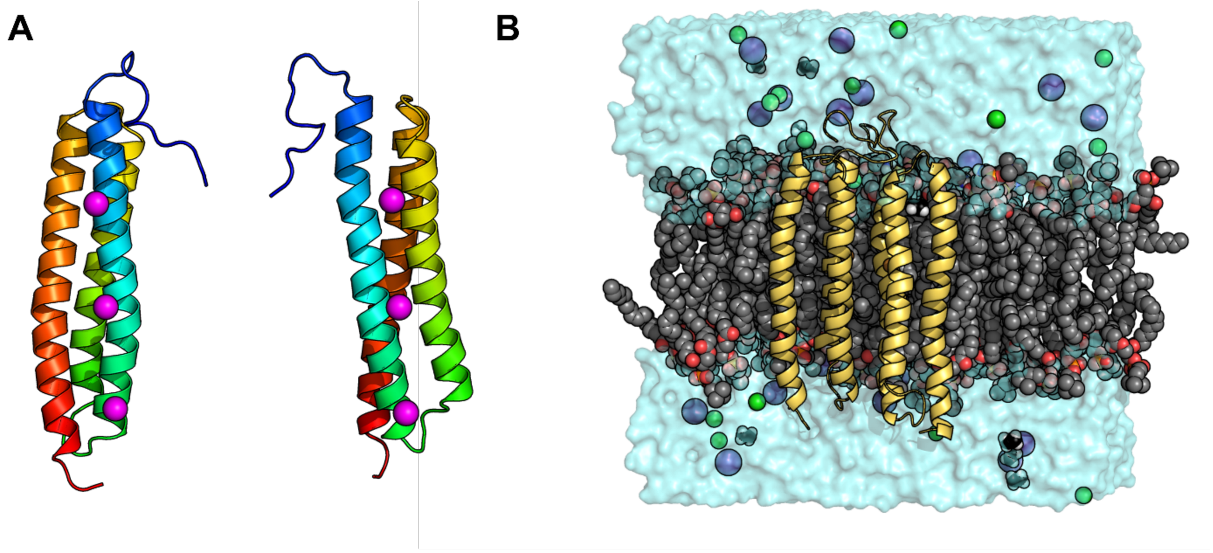

SI Figure 4. ETR1 membrane orientation. A) Selected monomer model, colored blue to red from the N-terminal to the C-terminal region, shown from two different orientations. The membrane orientation ("MEM" residue) obtained from Rosetta is superimposed in magenta spheres, where the central sphere represents the center of the membrane, and the external spheres represent the predicted upper and lower water-membrane interfaces. B) Representative snapshot of the dimer model in the molecular dynamics setup. All atoms in front of the protein were removed to allow a direct view onto the protein. The protein is shown in yellow, chloride ions in green, potassium ions in purple, and water as a transparent cyan surface. Ethylene and the aliphatic tails of the lipids are shown in black and grey, respectively. Hydrogens of the latter were omitted for clarity.

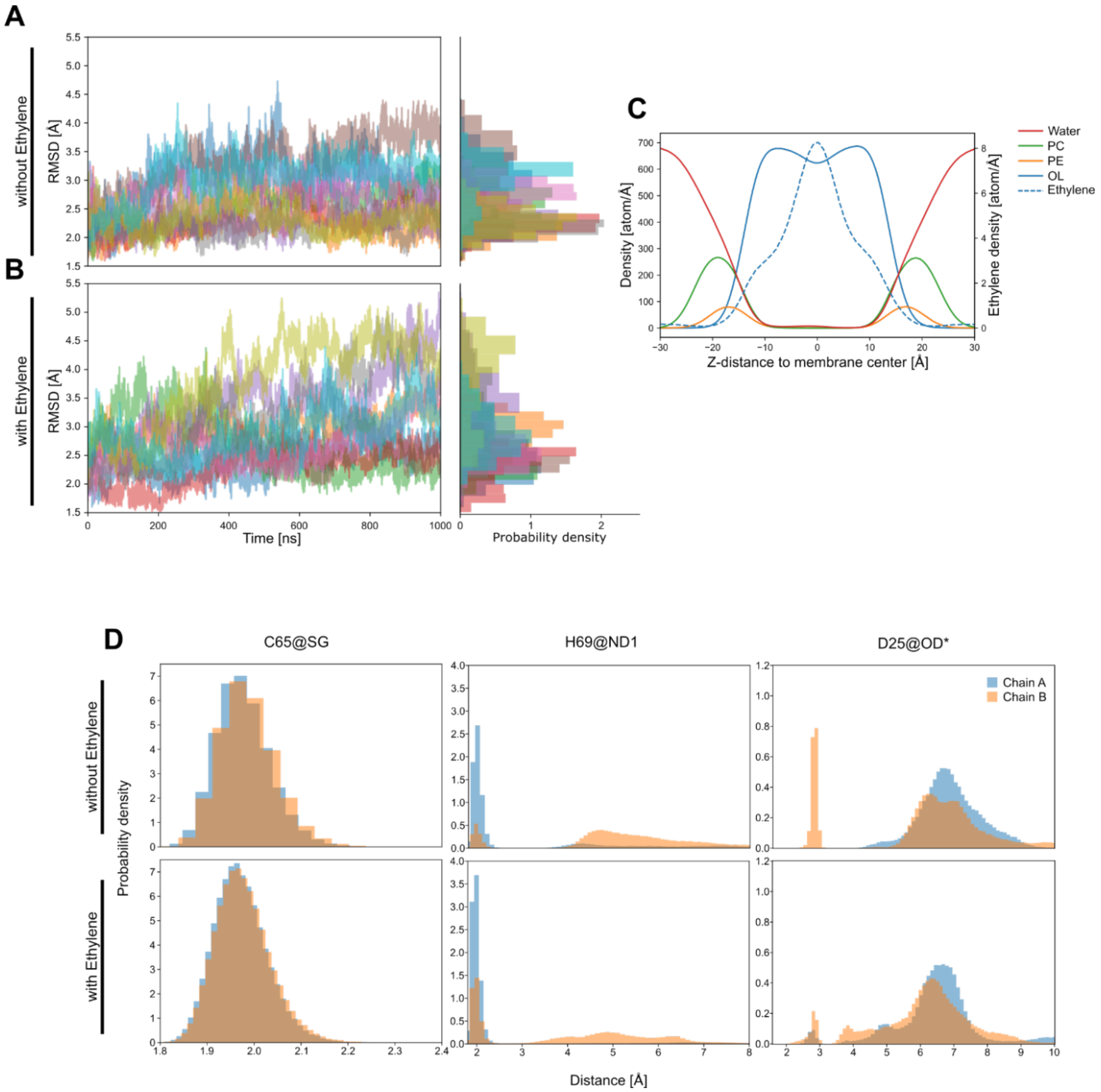

SI Figure 5. Analysis of MD simulations. A) & B) C_α RMSD for the simulated systems without (A) or with (B) ethylene with respect to the average structure. Residues 15-117 of both chains were considered for the calculation. Different colors represent different trajectories. On the right, normalized distributions of the time plots are shown. C) Decomposed density profile along the membrane axis. The plot shows the proportion of every component at a given distance of the membrane center. The dashed line shows the density for ethylene, with a peak at the center of the membrane slab. The ethylene molecules were added to the water bulk at the start and initially diffuse into the membrane during the simulations, from which they bind to the embedded dimer model of the ETR1 TMD. D) Histograms of distances between chelating atoms of C65, H69, or D25 and the Cu^{+} ion of the corresponding chain. Interactions with C65 prevail along the whole trajectory.

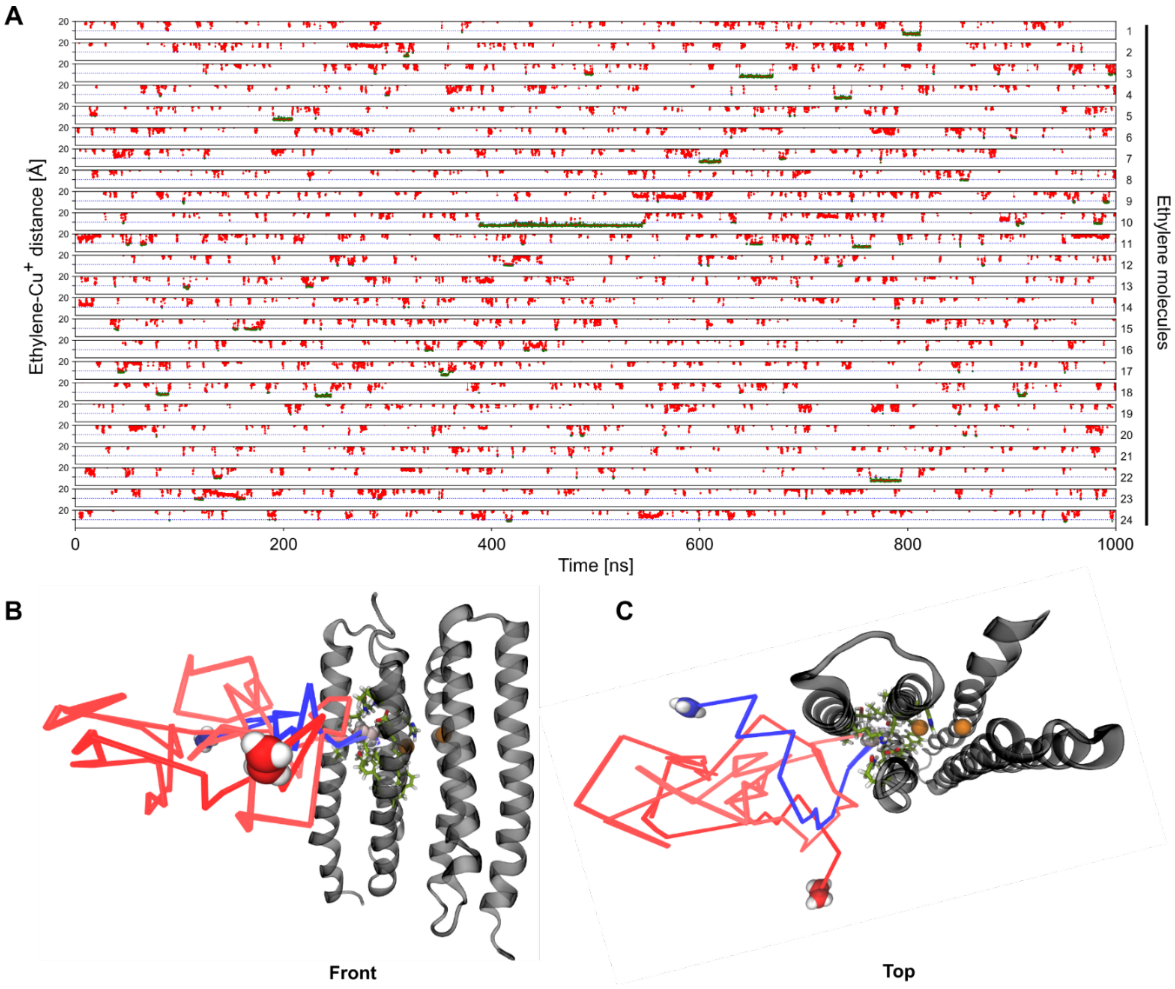

SI Figure 6. Ethylene binding during MD simulations of free ligand diffusion. A) Distance between the center of mass of the different ethylene molecules and one of the copper ions during the simulations. Only one representative replica and distances below 20 Å are shown. Distances below 10 Å are depicted in green to highlight binding events. B) and C) show the path of one representative binding event, with the panel in C rotated by 90 degrees with respect to the one in B). The coloring goes from red to blue, according to the simulation time. Binding site residues are depicted in green, while the Cu^{+} ions are depicted as orange spheres.

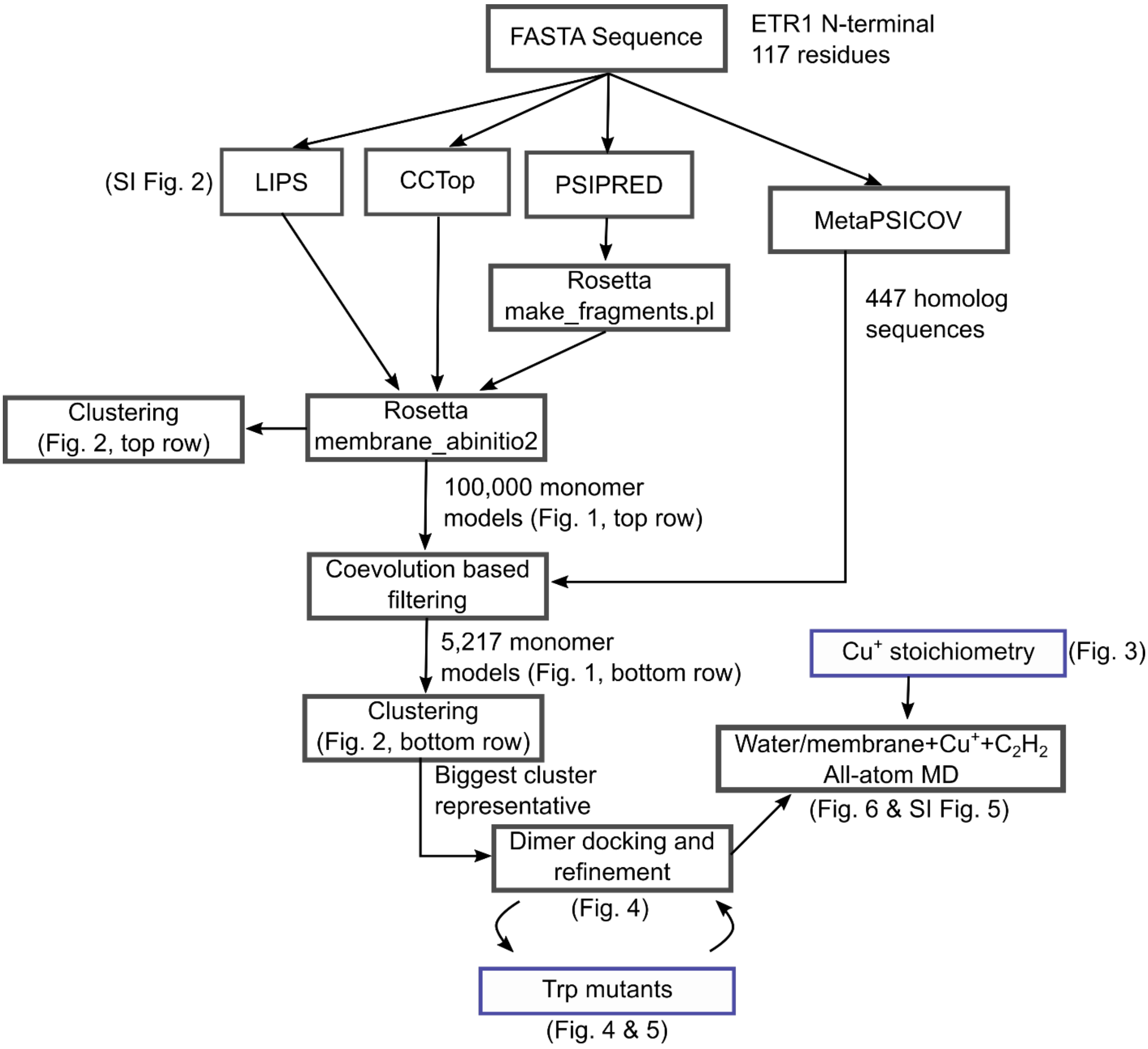

SI Figure 7. Summary of the modelling (black boxes) and experimental validation (blue boxes) process followed in this study. The main modelling tasks are depicted as a flowchart, with references to corresponding figures in the article. For details, refer to the main text.

4 Supplementary Tables

SI Table 1. Oligonucleotides used for cloning of ETR1 tryptophan mutants. Oligonucleotides were used for cloning of tryptophan mutants building megaprimer or by round-the-horn site-directed mutagenesis. Oligonucleotides used for round-the-horn site-directed mutagenesis were 5 prime phosphorylated.

**ETR1 tryptophan mutant**	**direction**	**Oligonucleotide**
ETR1^{W7X}_F26W	for	5'-GTAATTCATATGGAAGTCTGCAATTG-3'
	rev	5'-AATCGCAATGAACCAATCGGAGATGTA-3'
ETR1^{W7X}_F27W	for	5'-GTAATTCATATGGAAGTCTGCAATTG-3'
	rev	5'-CGCAATCGCAATCCAGAAATCGGAGAT-3'
ETR1^{W7X}_A29W	for	5'-ATTGCGTATTTTTCGATTCCTCTTGAGTTG-3'
	rev	5'-CCAAATGAAGAAATCGGAGATGTATTGGTA-3'
ETR1^{W7X}_F33W	for	5'-GTAATTCATATGGAAGTCTGCAATTG-3'
	rev	5'-AAGAGGAATCGACCAATACGCAATCGC-3'
ETR1^{W7X}_L39W	for	5'-GATTTACTTTGTGAAGAAATCAGCCGTGTT-3'
	rev	5'-CACTCAAGAGGAATCGAAAAATACGCAATC-3'
ETR1^{W7X}_V54W	for	5'-CTTGTTCAGTTTGGTGCTTTTATCGTTCTT-3'
	rev	5'-CCAAAATCTATACGGAAACACGGCTGATTT-3'
ETR1^{W7X}_L55W	for	5'-GTAATTCATATGGAAGTCTGCAATTG-3'
	rev	5'-ACCAAACTGAACCCATACAAATCTATA-3'
ETR1^{W7X}_F58W	for	5'-GTAATTCATATGGAAGTCTGCAATTG-3'
	rev	5'-GATAAAAGCACCCCACTGAACAAGTAC-3'
ETR1^{W7X}_L64W	for	5'-GTAATTCATATGGAAGTCTGCAATTG-3'
	rev	5'-AGTTGCTCCACACCAAACGATAAAAGC-3'
ETR1^{W7X}_T68W	for	5'-TGGCATCTTATTAACTTATTTACTTTCACT-3'
	rev	5'-TGCTCCACAAAGAACGATAAAAGC-3'
ETR1^{W7X}_L70W	for	5'-GTAATTCATATGGAAGTCTGCAATTG-3'
	rev	5'-AAATAAGTTAATCCAATGAGTTGCTCC-3'
ETR1^{W7X}_N72W	for	5'-TGGTTATTTACTTTCACTACGCATTCGA-3'
	rev	5'-AATAAGATGAGTTGCTCCACAAAGAACG-3'
ETR1^{W7X}_L73W	for	5'-GTAATTCATATGGAAGTCTGCAATTG-3'
	rev	5'-AGTGAAAGTAAACCAGTTAATAAGATG-3'
ETR1^{W7X}_T75W	for	5'-ATTAACTTATTTTGGTTCACTACGCATTCG-3'
	rev	5'-AAGATGAGTTGCTCCACAAAGAACG-3'
ETR1^{W7X}_A95W	for	5'-GTTGTCTCGTGTGCTACTGCGTTGAT-3'
	rev	5'-CCAGGTTAACACCTTCGCGGTAGTC-3'
ETR1^{W7X}_S98W	for	5'-GCTGTTGTCTGGTGTGCTACTGC-3'
	rev	5'-GGTTAACACCTTCGCGGTAGTCAT-3'
ETR1_TMD	for	5'-TAAGGATCCGGCTGCTAACA-3'
	rev	5'-TAAAGTGCTTCTAATCTCATGAGTC-3'

SI Table 2. Amounts of secondary structure in ETR1 tryptophan mutants determined by CD spectroscopy. The composition of structural elements in all constructed ETR1 tryptophan mutants were investigated by CD spectroscopy.

**ETR1 tryptophan mutant**	**α-helices [%]**	**β-strands [%]**	**β-turn [%]**	**Random coil [%]**
ETR1^{W7X}	33	18	21	28
ETR1^{W7X}_F26W	35	17	19	29
ETR1^{W7X}_F27W	34	18	20	28
ETR1^{W7X}_A29W	25	25	22	28
ETR1^{W7X}_F33W	35	18	19	28
ETR1^{W7X}_L39W	27	23	22	28
ETR1^{W7X}_V54W	22	27	23	28
ETR1^{W7X}_L55W	35	17	20	28
ETR1^{W7X}_F58W	36	18	19	27
ETR1^{W7X}_L64W	32	19	21	28
ETR1^{W7X}_T68W	30	20	23	27
ETR1^{W7X}_L70W	33	18	21	28
ETR1^{W7X}_N72W	31	19	21	29
ETR1^{W7X}_L73W	35	17	20	28
ETR1^{W7X}_T75W	27	21	23	29
ETR1^{W7X}_A95W	26	24	23	27
ETR1^{W7X}_S98W	29	20	22	29

5 Supplementary References

1 Mulnaes, D. & Gohlke, H. TopScore: Using Deep Neural Networks and Large Diverse Data Sets for Accurate Protein Model Quality Assessment. *J Chem Theory Comput* **14**, 6117-6126, doi:10.1021/acs.jctc.8b00690 (2018).

2 Forrest, L. R., Tang, C. L. & Honig, B. On the accuracy of homology modeling and sequence alignment methods applied to membrane proteins. *Biophys J* **91**, 508-517, doi:10.1529/biophysj.106.082313 (2006).

3 Crooks, G. E., Hon, G., Chandonia, J. M. & Brenner, S. E. WebLogo: a sequence logo generator. *Genome Res* **14**, 1188-1190, doi:10.1101/gr.849004 (2004).

4 Mol, A. R., S. Castro, M. & Fontes, W. NetWheels: A web application to create high quality peptide helical wheel and net projections. *bioRxiv*, doi:10.1101/416347 (2018).
